# Non-Isothermal Crystallization Behavior of a Zr-Based Amorphous Alloy Composite Prepared by Selective Laser Melting

**DOI:** 10.3390/ma18071631

**Published:** 2025-04-03

**Authors:** Qi An, Rui Li, Yalin Hu, Yun Luo, Anhui Cai, Yixian Li, Hong Mao, Sheng Li

**Affiliations:** 1 School of Information Science and Technology, Hunan Institute of Science and Technology, Yueyang 414000, China; anqi8643201@163.com; 2 School of Mechanical Engineering, Hunan Institute of Science and Technology, Yueyang 414000, China; 15115004137@163.com (R.L.); m18890409822@163.com (Y.H.); 15575025425@163.com (Y.L.); paomajun@163.com (H.M.); lishhnlg@hnist.edu.cn (S.L.)

**Keywords:** Zr-Cu-based amorphous alloys, selective laser melting, nonisothermal crystallization, crystallization mechanism

## Abstract

Zr_48_Cu_47.5_Al_4_Co_0.5_ bulk amorphous alloy composites were prepared by selective laser melting (SLM) technology under different processing conditions and their non-isothermal crystallization behaviors were systematically investigated. The results show that the crystallization phases are Cu_10_Zr_7_ and CuZr_2_ for both gas-atomized powder and SLMed samples. The dependence of volume fraction of Cu_10_Zr_7_ and CuZr_2_ on laser energy density can be fitted by an exponential function. The crystalline sizes of Cu_10_Zr_7_ and CuZr_2_ linearly increase with increasing energy density. The thermal stability is larger for the gas-atomized powders than for the SLMed bulk samples. It is interestingly found that there is an exponential relationship between the crystallization enthalpy Δ*H*_x_ and the amorphous content. In addition, the glass transition is more difficult for the gas-atomized powders than for the SLMed bulk samples. The crystallization procedure is more difficult for the SLMed bulk samples than for the gas-atomized powders. The local activation energy *E*_α_ decreases with increasing α for the gas-atomized powder and the SLMed bulk samples. In addition, the *E*_α_ is larger for the SLMed bulk samples than for the gas-atomized powder at the corresponding crystallization fraction α. The dependence of the local Avrami exponent n(α) on the α is similar for both the gas-atomized powders and the SLMed bulk samples at studied heating rates. The crystallization mechanism is also discussed.

## 1. Introduction

Zr-Cu-Al-Co amorphous alloys have intensively attracted attention due to their good glass-forming ability [1,2,3], high strength [2,3,4,5], good room-temperature plasticity and work-hardening [6,7,8], as well as good biocompatibility [3,7]. It has been found that Zr_55.8_Al_19.4_Co_17.36_Cu_7.44_ exhibited high glass-forming ability with a critical diameter of 10 mm and ultrahigh strength combined with large room-temperature plasticity [6]. Wu et al. [8] found that Zr_48_Cu_47.5_Al_4_Co_0.5_ alloy can be easily cast into bulk amorphous alloy composite and has high tensile strength and pronounced tensile ductility along with high work-hardening. However, the simple shapes and the limited size of the cast bulk amorphous alloys restricted the engineering application of the bulk amorphous alloys.

Additive manufacturing (AM) technology, known as three-dimensional (3D) printing, has rapidly developed due to its intriguing advantage of high cooling rates and achievement of arbitrary shapes. Thus, additive manufacturing technology can not only provide the opportunity to overcome the critical dimension limitation but also enable the forming of complex shapes for bulk amorphous alloys [9,10,11]. For example, Yang et al. [9] prepared a Zr_55_Cu_30_Ni_5_Al_10_ bulk amorphous alloy with complex geometries including cubic, hollow, and lattice structures by using a selective laser melting (SLM) technique and found that a milli/nano hierarchical porous structure through dealloying the lattice sample exhibits excellent catalytic properties towards degradation of methyl orange. A large number of experiments have been conducted on the preparation of bulk amorphous alloys or bulk amorphous alloy composites [12,13,14,15,16,17,18,19,20,21,22,23] through additive manufacturing. It has been found that the quality and the performances of the 3D-printed amorphous samples are affected by many factors, including laser related parameters (laser powder, spot size, pulse duration, pulse frequency), scan related parameters (scan speed, scan spacing, scan patterns), powder related parameters (particle size, particle shape and distribution, powder bed density, layer thickness, materials property), and temperature related parameters (powder bed temperature, powder feeder temperature, temperature uniformity), resulting in different structures and properties of the 3D-printed amorphous alloys [9,10,11,12,13,14,15,16,17,18,19,20,21,22,23]. Liu et al. [15] critically reviewed the pores and cracks in the laser powder bed fusion (LPBF) fabricated MGs, and the pores and cracks initiation and alleviation mechanisms were further analyzed. Li et al. [18] systematically investigated the effects of laser power and scanning speed within a large processing window on the density, structure and mechanical properties from two different starting powders of Zr_59.3_Cu_28.8_Nb_1.5_Al_10.4_ amorphous alloy powder by using laser powder bed fusion (LPBF). They obtained the optimal energy density regions for the LPBFed samples with high relative densities (>99%) for coarse powder with low oxygen and fine powder with higher oxygen and found that the strength and hardness for the fully XRD amorphous samples increased with increasing energy density, while the relaxation enthalpy and ductility decreased. In addition, it is well known that the properties of amorphous alloy composites are related to the size, category, distribution, and orientation of the crystalline phases. The crystalline phases usually exist in the 3D-printed amorphous alloys and deteriorate mechanical properties of the 3D-printed bulk amorphous alloy composites [9,18,19,20,21]. The crystallization behavior has been intensively investigated for amorphous alloys during 3D-printing procedures [9,18,19,20], while it has sparsely been discussed for 3D-printed amorphous alloys during service or processing [21,22]. For example, Pacheco et al. [21] investigated thermal stability by using the DSC technique at 20 K/min and the crystallization phase precipitation behavior by using in situ synchrotron radiation XRD for SLMed Zr_59.3_Cu_28.8_Al_10.4_Nb_1.5_ amorphous alloy. However, the nonisothermal crystallization mechanism was not a concern. Ouyang et al. [22] investigated the isothermal crystallization behavior of 3D-printed Zr_55_Cu_30_Ni_5_Al_10_ bulk amorphous alloy and found that there are four crystallization processes corresponding to different regions. Thus, it is crucial for the investigation of the crystallization behavior of 3D-printed amorphous alloys due to the thermodynamic instability of amorphous alloys.

Moreover, Zhang et al. [23] recently prepared a Zr_47.5_Cu_45.5_Al_5_Co_2_ bulk amorphous alloy composite containing a nearly B2-ZrCu single phase using the LPBF technique and found that the B2 phase reinforced bulk amorphous alloy composite exhibited excellent mechanical properties with enhanced plasticity and toughness. They also found that the mechanical properties of the B2 phase reinforced bulk amorphous alloy composite can be easily modulated by altering the amount of the B2 phase via adjusting the laser energy input. Gao et al. [24] successfully fabricated a crack-free Zr_47_Cu_46_Al_6_Co_1_ amorphous alloy composite with a B2-ZrCu single phase by selective laser melting and found that the microstructure is sensitive to processing parameters. The composites showed high fracture strength and anisotropic mechanical property. Kozachkov et al. [25] investigated the effect of cooling rate on the volume fraction of the B2 ZrCu phase in Zr_48_Cu_47.5_Al_4_Co_0.5_ amorphous alloy composites and found that the B2 ZrCu phase conglomerated into large areas and the amorphous matrix no longer percolated through the sample when the volume fraction of B2 ZrCu crystals exceeded 30%. Beyond this percolation threshold, the mechanical behavior has shown to switch from rule-of-mixtures to a load-bearing type response, resulting in profound degradation of yield strength. It is also well known that the performance of amorphous alloys can be modified by heat processing technology. However, the crystallization behavior and especially the crystallization mechanism for these Zr-Cu based 3D-printed amorphous alloy composites was not involved to our best knowledge.

Therefore, in the present work, gas-atomized Zr_48_Cu_47.5_Al_4_Co_0.5_ amorphous alloy powder whose composition is similar to the above-mentioned Zr-Cu-Al-Co alloys was selected to prepare the bulk amorphous alloy by using the SLM technique under different processing parameters. The crystallization behavior was systematically investigated, and the crystallization mechanism was also discussed under nonisothermal mode.

## 2. Experimental

The gas-atomized Zr_48_Cu_47.5_Al_4_Co_0.5_ amorphous alloy powders with 99.95% purity were purchased from Peshing new metal (Changzhou) Co. Ltd., Changzhou, China. The size of the used alloy powders is 15~53 μm, which is suitable for 3D-printing. The detailed morphology of the amorphous alloy powders is shown in our previous work [26].

The bulk amorphous alloy composites were prepared by selective the laser melting (SLM) technique using an iSLM280 printer supplied by Suzhou ZRapid Tech, Suzhou, China. The detailed SLM parameters in the present work are listed in Table 1. The forming chamber was vacuumed and protected by argon gas with 99.99% purity. The chamber pressure was maintained to be 0.04 KPa. The 304 stainless steel substrate was preheated up to 60 °C. The laser power *P* was selected as 75~90 W. The laser exposure time was 100 μs with a halting time of 10 μs. The hatch spacing *h* was 0.03 mm. The powder layer thickness *d* was 0.08~0.1 mm. The laser scanning velocity *v* was 1500~2500 mm/s. The volume energy density *E_V_* of each sample can be calculated as $E_{v}=\frac{P}{vhd}$ [13].

The amorphous structures of the as-received alloy powder and the SLMed bulk alloys were identified by X-ray diffraction (XRD) using an XD-3A diffractometer with Cu-K_α_. Jade 9 software was applied for the determination of type, fraction, and size of the crystallization phases. DSC was used to investigate nonisothermal crystallization behavior with a DSC-3 differential scanning calorimeter (DSC) at 10~40 K/min heating rates under a flowing argon atmosphere with 99.999% purity.

## 3. Results

**Figure 1:**
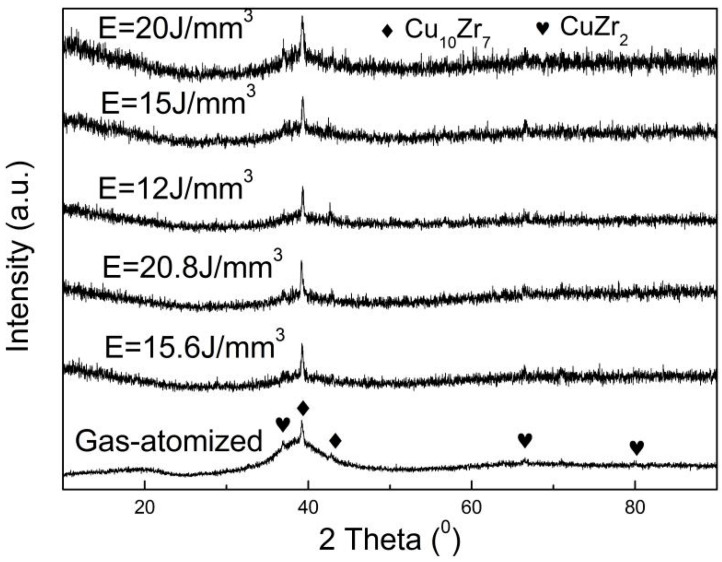
XRD patterns for gas-atomized powder and 3D-printed bulk amorphous samples under different energy density.

Figure 1 shows XRD patterns for the gas-atomized powders and the 3D-printed bulk samples under different conditions. The Bragg diffraction patterns for the gas-atomized powder and the 3D-printed bulk samples are the same as each other. The Bragg diffraction peaks can be perfectly indexed by Cu_10_Zr_7_ and CuZr_2_ phases, which is similar with the results of the Zr_48_Cu_47.5_Al_4_Co_0.5_ bulk amorphous alloys prepared by spark plasma sintering (SPS) [26]. It has been found that the Zr_48_Cu_47.5_Al_4_Co_0.5_ alloy is usually cast into B2 CuZr phase reinforced amorphous alloy composites [8,24,25]. The crystalline phase is a B2 CuZr phase for 3D-printed Zr-Cu based bulk amorphous alloys (Zr_47.5_Cu_45.5_Al_5_Co_2_ [23], Zr_47_Cu_46_Al_6_Co_1_ [24]). However, Han et al. [27] found that the crystallization phases are Cu_10_Zr_7_, CuZr_2_, and τ4 in fully crystallized Zr_48_Cu_47.5_Al_4_Co_0.5_ amorphous alloy when the heating rate is less than 2 K/s. Lan et al. [28] found that Zr_48_Cu_45_Al_7_ bulk amorphous alloy was crystallized into two-phase coexistence of Cu_10_Zr_7_ and CuZr_2_. The B2 CuZr phase is a well-known high-temperature unstable phase and can be decomposed into low-temperature equilibrium phases of Cu_10_Zr_7_ and CuZr_2_ when the cooling rate is low enough [25].

The volume fraction and crystal size for the two crystalline phases were estimated by using Jade 9 software and are listed in Table 2. Both volume fraction and size of Cu_10_Zr_7_ and CuZr_2_ phases for the 3D-printed samples are larger than those for the gas-atomized powders. In addition, both volume fraction and size of Cu_10_Zr_7_ and CuZr_2_ phases increase with increasing volume energy density, which is similar to the results of the Zr_48_Cu_47.5_Al_4_Co_0.5_ bulk amorphous alloy composites using spark plasma sintering (SPS) [26].

**Figure 2:**
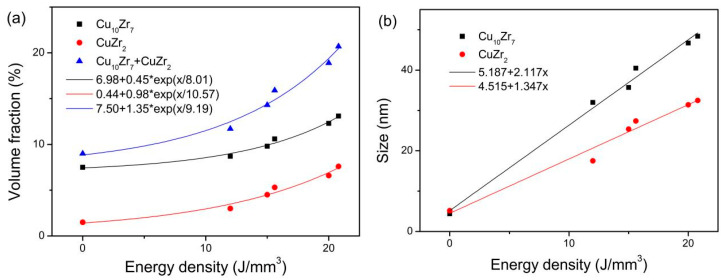
Dependence of volume fraction (**a**) and size (**b**) of Cu_10_Zr_7_ and CuZr_2_ on energy density.

As shown in Figure 2a, the dependence of volume fraction of Cu_10_Zr_7_ and CuZr_2_ on volume energy density can be fitted by using an exponential function. The fitting results are *y* = 6.98 + 0.45exp(*x*/8.01) for Cu_10_Zr_7_ and *y* = 0.44 + 0.98exp(*x*/10.57) for CuZr_2_, respectively. The growth rate of volume fraction is larger for CuZr_2_ than for Cu_10_Zr_7_. In addition, the total volume fraction of the crystalline phases (Cu_10_Zr_7_ + CuZr_2_) exponentially changes with the volume energy density, i.e., *y* = 7.50 + 1.35exp(*x*/9.19). However, Cai et al. [26] found that the volume fraction of Cu_10_Zr_7_ and CuZr_2_ of the SPSed Zr_48_Cu_47.5_Al_4_Co_0.5_ bulk amorphous alloy composites linearly increases with increasing sintering temperatures. The growth rate of volume fraction is larger for Cu_10_Zr_7_ than for CuZr_2_. It has been found that there are exponential relationships between the fraction of the crystalline phase and the cooling rate for Mg_65_Zn_30_Ca_5_ amorphous alloy composite [29], between the crystallization fraction and the 3D-printed parameters (such as beam diameter, dense layer thickness, preheating temperature, and waiting time) for Zr_59.3_Cu_28.8_Al_10.4_Nb_1.5_ amorphous alloy composite [30], and between the crystallized volume fraction and the time delay for 3D-printed Zr_59.3_Cu_28.8_Al_10.4_Nb_1.5_ bulk amorphous alloy composites [31]. On the other hand, it can be clearly observed from Figure 2b that the dependence of size of Cu_10_Zr_7_ and CuZr_2_ on energy density can be fitted by a linear function. The fitting results are *y* = 5.187 + 2.117*x* for Cu_10_Zr_7_ and *y* = 4.515 + 1.347*x* for CuZr_2_, respectively. The growth rate of size is larger for Cu_10_Zr_7_ than for CuZr_2_. Cai et al. [26] found that the size of Cu_10_Zr_7_ and CuZr_2_ of the SPSed Zr_48_Cu_47.5_Al_4_Co_0.5_ bulk amorphous alloy composites linearly increase with increasing sintering temperatures. The growth rate of the size is larger for CuZr_2_ than for Cu_10_Zr_7_. Li et al. [18] found that the crystal size increased with increasing energy density for the 3D-printed Zr_59.3_Cu_28.8_Al_10.4_Nb_1.5_ bulk amorphous alloy composites.

**Figure 3:**
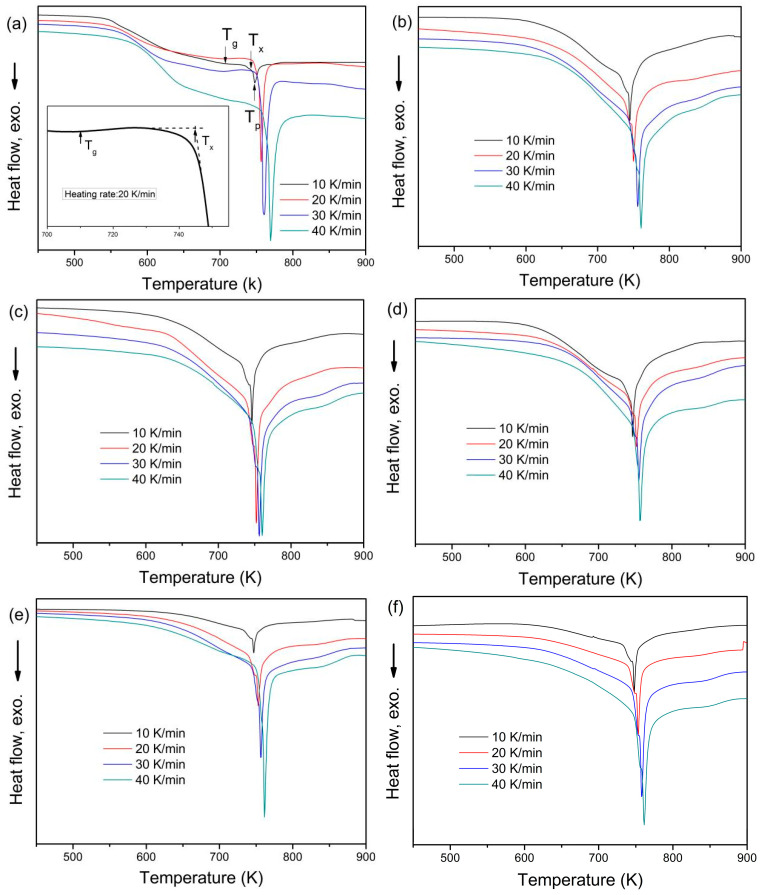
DSC traces for gas-atomized powders (**a**) and 3D-printed bulk samples under different energy density: 15.6 J/mm^3^ (**b**), 20.8 J/mm^3^ (**c**), 12 J/mm^3^ (**d**), 15 J/mm^3^ (**e**), and 20 J/mm^3^ (**f**), respectively.

**Figure 4:**
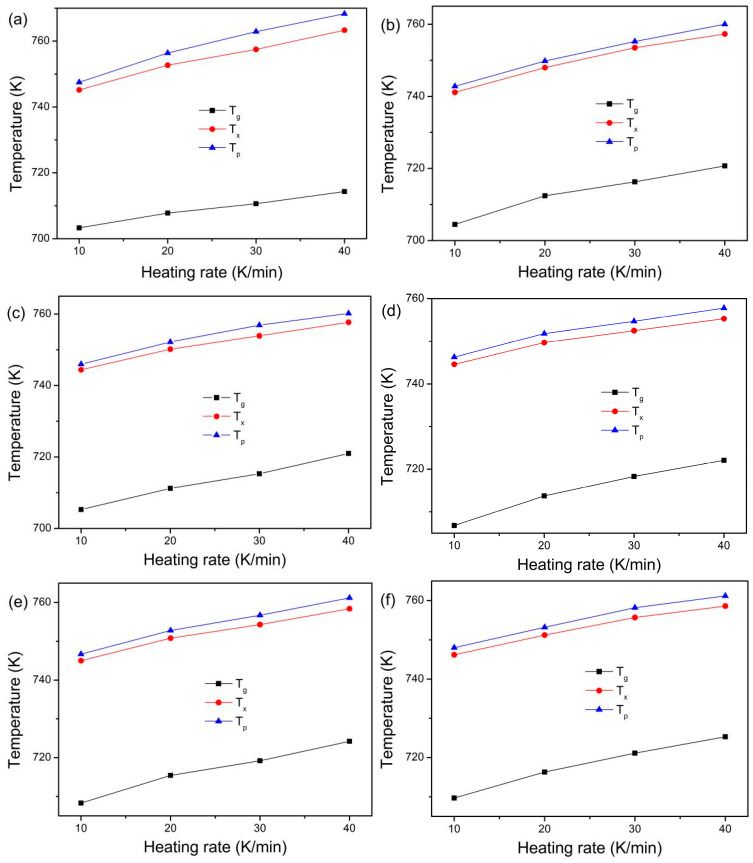
Dependence of characteristic temperatures (*T*_g_, *T*_x_, and *T*_p_) for gas-atomized powders (**a**) and 3D-printed bulk samples under different energy density: 15.6 J/mm^3^ (**b**), 20.8 J/mm^3^ (**c**), 12 J/mm^3^ (**d**), 15 J/mm^3^ (**e**), and 20 J/mm^3^ (**f**), respectively.

The crystallization behavior for the gas-atomized powder and the 3D-printed bulk samples was further investigated by non-isothermal mode at 10~40 K/min heating rates, respectively. The DSC traces are shown in Figure 3. Obviously, there is an endothermic peak for the glass transition procedure, and then following, an exothermic peak for the crystallization procedure. The characteristic temperatures, including glass transition temperature *T*_g_,, onset crystallization temperature *T*_x_, and crystallization peak temperature *T*_p_ are shown in Figure 3, and the detailed data are presented in Figure 4. These characteristic temperatures all increase with the increase of the heating rates for both gas-atomized powders and 3D-printed bulk samples. It is noted that these characteristic temperatures are very close to each other for both gas-atomized powders and 3D-printed bulk samples under the corresponding heating rate. In addition, the undercooled liquid region Δ*T*_x_ (=*T*_x_ − *T*_g_) was calculated and listed in Table 3. Clearly, the Δ*T*_x_ is larger for the gas-atomized powders than for the 3D-printed bulk samples, indicating that the thermal stability is larger for the former than for the latter. Frey et al. [19] found that both *T*_g_ and *T*_x_ are larger for the 3D-printed bulk amorphous alloy composites than for the corresponding alloy powders for Vit101 and Vit101Si. Sohrabi et al. [31] found that both *T*_g_ and *T*_x_ are smaller for the 3D-printed Zr_59.3_Cu_28.8_Al_10.4_Nb_1.5_ bulk amorphous alloy composites than for the corresponding amorphous alloy powder, while on the contrary, for the Δ*T*_x_, Li et al. [18] found that the *T*_x_ for the 3D-printed Zr_59.3_Cu_28.8_Al_10.4_Nb_1.5_ bulk amorphous alloys does not depend on the energy density and is larger for the 3D-printed samples using fine powders and smaller for the 3D-printed samples using coarse powders than the corresponding amorphous alloy powders. Pacheco et al. [20] investigated thermal stability and crystallization of Zr_59.3_Cu_28.8_Al_10.4_Nb_1.5_ amorphous alloy produced by suction casting and selective laser melting and found that the *T*_g_ was smaller for the SLM sample than for the cast sample, while on the contrary, for both *T*_x_ and Δ*T*_x_, Yang et al. [9] found that the *T*_g_ was larger for the 3D-printed Zr_55_Cu_30_Ni_5_Al_10_ sample than for the corresponding alloy powder.

**Figure 5:**
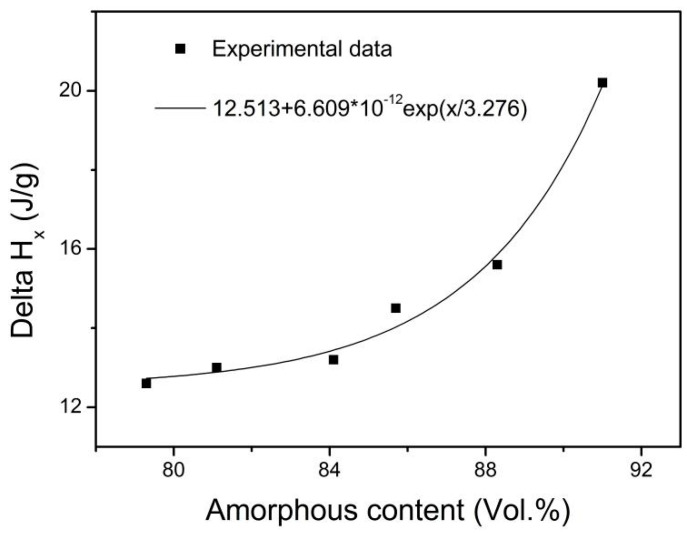
Dependence of Δ*H*_x_ on amorphous content.

Moreover, the crystallization enthalpy Δ*H*_x_ was also calculated and listed in Table 3. The Δ*H*_x_ decreases with increasing energy density. Sohrabi et al. [31] found that the Δ*H*_x_ is smaller for the 3D-printed Zr_59.3_Cu_28.8_Al_10.4_Nb_1.5_ bulk amorphous alloy composites than for the corresponding amorphous alloy powder. Yang et al. [9] found that the Δ*H*_x_ was smaller for a 3D-printed Zr_55_Cu_30_Ni_5_Al_10_ sample than for the corresponding alloy powder. It is well known that the larger the Δ*H*_x_, the more amorphous the content. The dependence of Δ*H*_x_ on amorphous content is presented in Figure 5. It is interestingly found that there is not a linear but an exponential relationship between Δ*H*_x_ and amorphous content in the present work. The fitting equation is *y* = 12.513 + 6.609 × 10^−12^exp(*x*/3.276).

**Figure 6:**
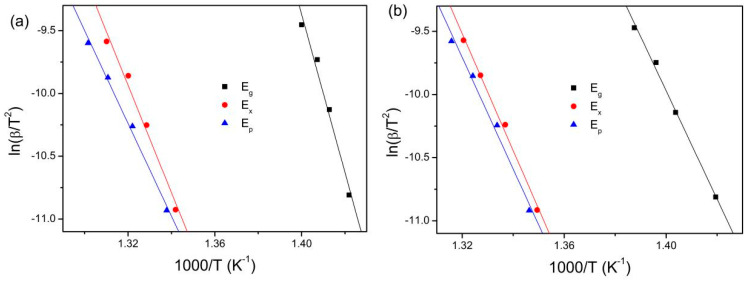

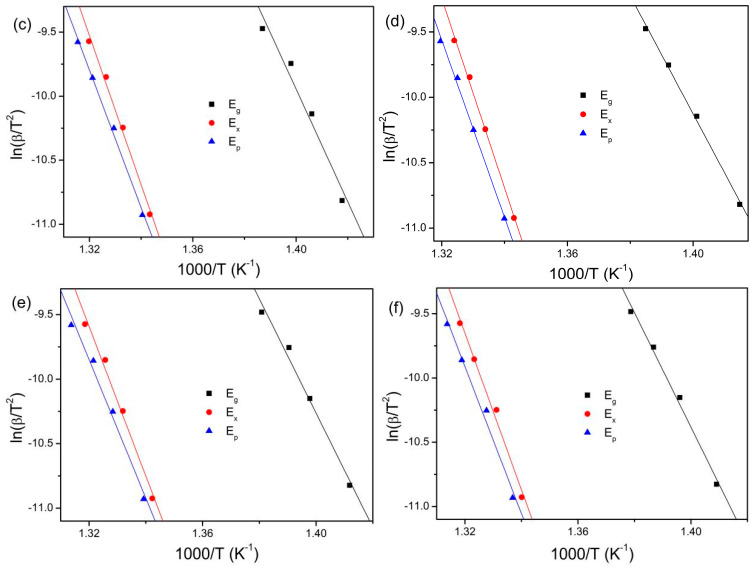
Kissinger plots for gas-atomized powders (**a**) and 3D-printed bulk samples under different energy densities: 15.6 J/mm^3^ (**b**), 20.8 J/mm^3^ (**c**), 12 J/mm^3^ (**d**), 15 J/mm^3^ (**e**), and 20 J/mm^3^ (**f**), respectively.

The activation energies for glass transition *E*_g_, onset crystallization *E*_x_, and crystallization peak *E*_p_ were estimated by Kissinger equation [32]: ln(β/T^2^) = *E*/RT + constant. Where β is the heating rate, R is the gas constant, and *T* is *T*_g_, *T*_x_, and *T*_p_, respectively. The Kissinger plots for the gas-atomized powders and the 3D-printed bulk samples are presented in Figure 6. The calculated *E*_g_, *E*_x_ and *E*_p_ are listed in Table 3. The sequence of the activation energies is *E*_g_ > *E*_x_ > *E*_p_ for the gas-atomized powder, suggesting the glass transition is more difficult than the crystallization procedure, which is similar with the results for (Zr_47_Cu_46_Al_7_)_97_Ti_3_ [33], Zr_48_Cu_43_Al_9_ [34], (Zr_48_Cu_43_Al_9_)_98_Y_2_ [34] and Zr-Al-Ni-Cu amorphous alloys (Zr_65_Al_8_Ni_8.5_Cu_18.5_, Zr_65.5_Al_7.3_Ni_7.35_Cu_19.85_ and Zr_66_Al_6.6_Ni_6.2_Cu_21.2_) [35], and Zr_51_Al_12_Ti_18_Cu_19_ and Zr_51_Al_16_Ti_14_Cu_19_ amorphous alloy composite powders [36]. However, the sequence of the activation energies is *E*_x_ > *E*_p_ > *E*_g_ for the 3D-printed bulk samples, indicating that the crystallization procedure is more difficult than the glass transition, which is similar with the results of Cu-Zr-Ti amorphous alloy composite powders [37] and Cu_50_Zr_40_Ti_10_ amorphous alloy powders [38]. Moreover, the *E*_g_ is larger for the gas-atomized powder than for the 3D-printed bulk samples. This indicates that the glass transition is more difficult for the former than for the latter. Both *E*_x_ and *E*_p_ are larger for the 3D-printed bulk samples than for the gas-atomized powder. This implies that the crystallization procedure is more difficult for the former than for the latter.

**Figure 7:**
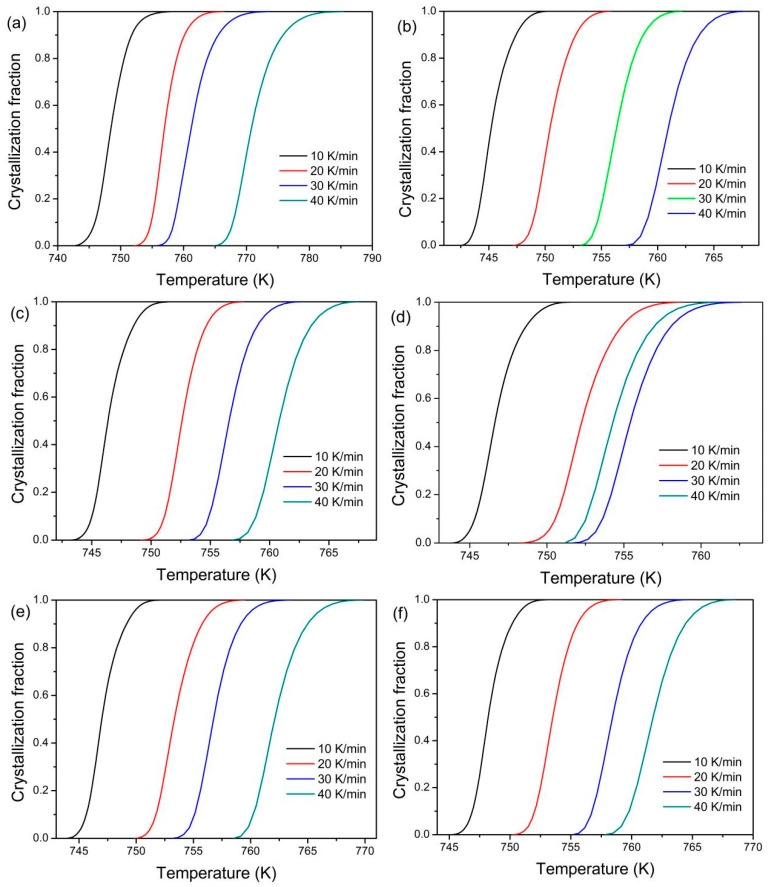
Relationships between crystallization fraction and temperature for gas-atomized powders (**a**) and 3D-printed bulk samples under different energy densities: 15.6 J/mm^3^ (**b**), 20.8 J/mm^3^ (**c**), 12 J/mm^3^ (**d**), 15 J/mm^3^ (**e**), and 20 J/mm^3^ (**f**), respectively.

The local crystallization activation energy *E*_α_ is estimated to analyze the crystallization kinetics during the whole crystallization procedure at different crystallization fractions. The crystallization fraction α is calculated by α = A_T_/A. A_T_ is the area of the crystallization procedure from the onset crystallization temperature to a given temperature, and A is the area of the whole crystallization procedure. The relationships between the crystallization fraction and the temperature at different heating rates are shown in Figure 7. All curves show the typical sigmoid shape and shift towards high temperatures with the increase of the heating rate.

**Figure 8:**
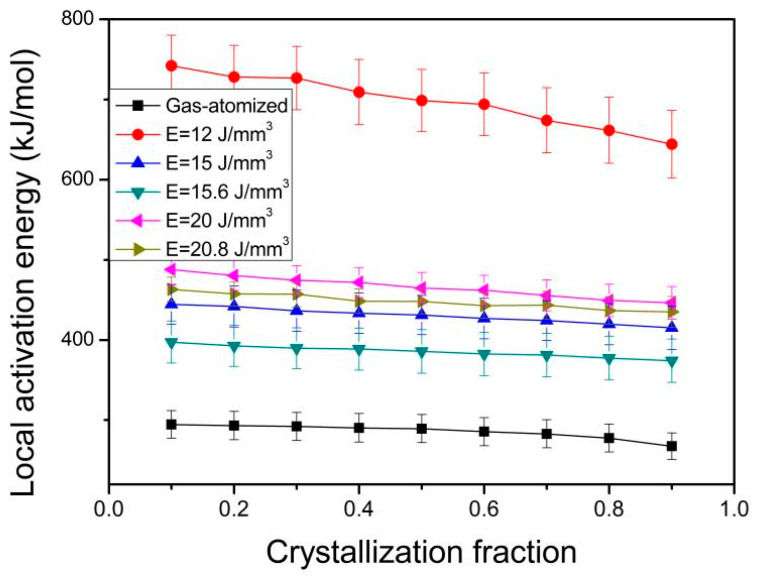
Relationships between crystallization fraction and local activation energy for gas-atomized powders and 3D-printed bulk samples under different energy densities.

The *E*_α_ is calculated from the fitting straight line by plotting ln(β) vs. 1000/T_α_ at a given α [39]. Figure 8 presents the dependence of the *E*_α_ on the α. As can be observed from Figure 8, the *E*_α_ for the gas-atomized powder and the 3D-printed bulk samples decreases with increasing α, which is similar with the nonisothermal crystallized results of Zr_51_Al_16_Ti_14_Cu_19_ amorphous alloy composite powder [36] and (Zr_54_Al_10.2_Ni_9.4_Cu_26.4_)_100−x_Ti_x_ (x = 0–0.5 at.%) bulk amorphous alloys [40], while opposite to Zr_51_Al_12_Ti_18_Cu_19_ amorphous alloy composite powders [36]. In addition, similar isothermal crystallized dependences of the *E*_α_ on the α were found for Zr-Al-Ni-Cu bulk amorphous alloys (Zr_65_Al_8_Ni_8.5_Cu_18.5_, Zr_65.5_Al_7.3_Ni_7.35_Cu_19.85_, and Zr_66_Al_6.6_Ni_6.2_Cu_21.2_) [35], Zr_59.3_Cu_28.8_Al_10.4_Nb_1.5_ amorphous alloy powders [41], Zr_46_Cu_38_Ag_8_Al_8_ [42], Zr_50_Cu_50_ [43], Zr_55_Cu_30_Ni_5_Al_10_ [44,45], and (Zr_55_Cu_30_Al_10_Ni_5_)_98_Nb_2_ amorphous alloys [44], while opposite to Zr_51_Al_12_Ti_18_Cu_19_ and Zr_51_Al_16_Ti_14_Cu_19_ amorphous alloy composite powders [36]. In addition, the *E*_α_ is larger for the 3D-printed bulk samples than for the gas-atomized powder at a given α. As for the 3D-printed bulk samples, the *E*_α_ is the largest for the bulk sample in 12 J/mm^3^ and the smallest for the one in 15.6 J/mm^3^. The averaged *E*_α_ is 286.0 kJ/mol for the gas-atomized powder, 385.5 kJ/mol for 15.6 J/mm^3^, 448.1 kJ/mol for 20.8 J/mm^3^, 697.6 kJ/mol for 12 J/mm^3^, 430.3 kJ/mol for 15 J/mm^3^, and 465.9 kJ/mol for 20 J/mm^3^, respectively. These results are in well agreement with the crystallization activation energies in Table 3. The crystallization activation energies are 438~448 kJ/mol for Zr_50_Cu_50_ amorphous alloy and 370~418 kJ/mol for (Zr_50_Cu_50_)_100−x_Al_x_ (x = 5–8 at.%) amorphous alloys in non-isothermal heating mode [46]. Qiao et al. [47] found that the average crystallization activation energies for Cu_46_Zr_45_Al_7_Y_2_ bulk metallic glass are 316 kJ/mol in non-isothermal heating mode and 484 kJ/mol in isothermal heating mode.

**Figure 9:**
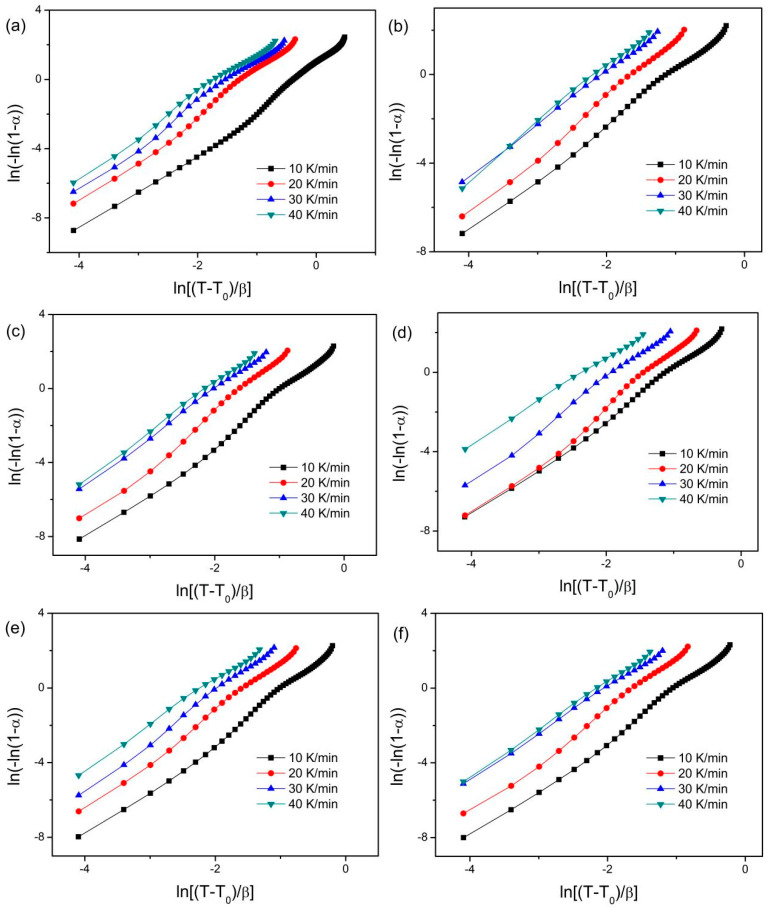
Relationship between ln(−ln(1 − α)) and ln[(T − T_0_)/β] at different heating rates. (**a**) gas-atomized powder, (**b**) E = 15.6 J/mm^3^, (**c**) E = 20.8 J/mm^3^, (**d**) E = 12 J/mm^3^, (**e**) E = 15 J/mm^3^, and (**f**) E = 20 J/mm^3^, respectively.

Moreover, the local Avrami exponent n(α) is estimated for clarifying the crystallization mechanism. The n(α) is calculated by the extended JMA method [48]: $n(\alpha )=B\frac{d\ln[-\ln(1-\alpha )]}{d[(T-T_{0})/\beta ]}$ and $B=\frac{1}{1+E/R(T-T_{0})}$. Where *T*_0_ is the onset crystallization temperature and *E* is the activation energy. Figure 9 shows the relationship between ln[−ln(1 − α)] and (T − T_0_)/β. The n(α) values can be estimated by the slope of the plots between ln[−ln(1 − α)] and (T − T_0_)/β. It can be clearly observed from Figure 9 that there are not linear relationships between ln[−ln(1 − α)] and (T − T_0_)/β, indicating the dependence of the n(α) on the α. The non-linear Avrami plots have been reported for the crystallization process of various amorphous alloys or metallic glasses [35,36,41,42,43,44,45,47], which would be due to the non-homogeneous distribution of the nuclei as the crystallization fraction increases [49], resulting in a change of the crystallization mechanism.

**Figure 10:**
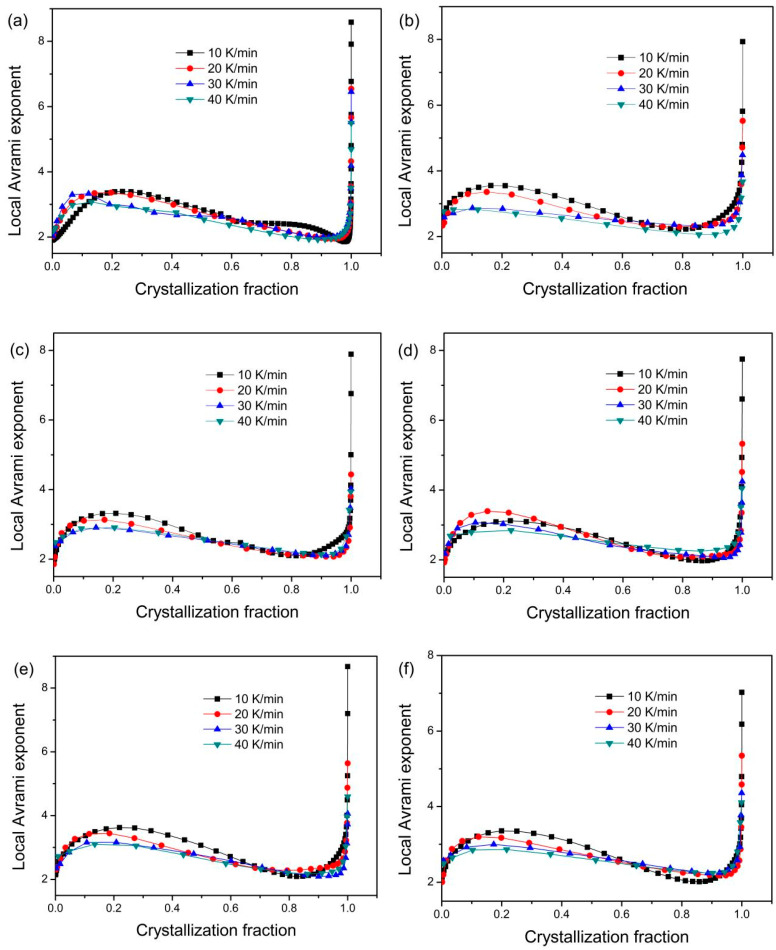
Relationship between n(α) and α at different heating rates. (**a**) gas-atomized powder, (**b**) E = 15.6 J/mm^3^, (**c**) E = 20.8 J/mm^3^, (**d**) E = 12 J/mm^3^, (**e**) E = 15 J/mm^3^, and (**f**) E = 20 J/mm^3^, respectively.

The dependences of the n(α) on the α are presented in Figure 10 for the gas-atomized powder and the 3D-printed bulk samples. Obviously, all curves are almost similar to each other at different heating rates. The n(α) firstly increases and then decreases with increasing α, then finally sharply increases at the end of the crystallization process. The increase of n(α) at the initial crystallization stage would be that the pre-existing nuclei/crystal trigger the nucleation of the surrounding regions, resulting in the increase of nucleation/growth rate. As crystallization fraction increases, long-range diffusion of atoms is difficult, which retards the nucleation and growth, resulting in decreasing nucleation/growth rate. The growth of the initial nuclei changes the composition of the matrix surrounding the nuclei. The compositional fluctuation facilitates the nucleation adjacent to the growing nuclei, leading to a higher nucleation rate compared to that at the initial crystallization stage. These results indicate that the nucleation and/or growth is related to the α. The dependences of the n(α) on the α in the present work are similar to the nonisothermal crystallized results of (Zr_54_Al_10.2_Ni_9.4_Cu_26.4_)_100−x_Ti_x_ (x = 0–0.5 at.%) bulk amorphous alloys [40] and the isothermal crystallized results of Zr_50_Cu_50_ [43], Zr_46_Cu_38_Ag_8_Al_8_ [45], and Cu-Zr-Ti [37] amorphous alloys.

## 4. Discussion

As shown in Figure 1, the Cu_10_Zr_7_ and CuZr_2_ phases simultaneously exist in the SLMed bulk samples and the amorphous alloy powder. According to the Cu-Zr binary phase diagram [50], there is an eutectoid transformation, i.e., CuZr→Cu_10_Zr_7_ + CuZr_2_, for the alloys close to the Zr_50_Cu_50_ (at.%) composition. Thus, when the cooling rate during the solidification process of the alloy melting close to Zr_50_Cu_50_ is too low to obstruct the precipitation of phases, the ZrCu phase’s even coexistence of Cu_10_Zr_7_ and CuZr_2_ can usually be observed [8,24,25]. Especially, the 3D-printing procedure is a process of repeated heating and solidification, resulting in the formation of the heat-affected zone (HAZ). When the laser energy density is high enough, the temperature of the HAZ can reach up to the crystallization temperature (*T*_x_), leading to the crystallization of the HAZ. It has been found that Zr_48_Cu_47.5_Al_4_Co_0.5_ amorphous alloy can crystallize into Cu_10_Zr_7_, CuZr_2_, and τ4 [27]. Lan et al. [28] found that Zr_48_Cu_45_Al_7_ bulk amorphous alloy crystallized into the coexistence of Cu_10_Zr_7_ and CuZr_2_.

On the other hand, the fraction of crystallization phases or amorphous phase increases with increasing energy density. The reason would be as follows. It is well known that the crystallization of amorphous alloys or metallic glasses originates from the HAZ during the 3D-printing procedure. In addition, it has been found that the critical heating rate is much higher than the critical cooling rate for avoiding crystallization of the amorphous alloys [51]. The heating rate as well as the cooling rate are both critical for crystallization in the HAZ [52]. Kim et al. [52] investigated the phase evolution in Cu_54_Ni_6_Zr_22_Ti_18_ bulk metallic glass Nd:YAG laser welds and found that the cooling rate decreases as pulse energy increases [52]. Sun and Flores found that the depth of the melt zone and thickness of the HAZ increase as the energy density increases [53]. Numerous studies have found that the amorphous content decreases with increasing energy density for metallic glass fabricated by selective laser melting [54,55]. The higher energy density not only brings more energy to fuse the powder particles but also induces stronger in situ heat treatment and consequently more crystallization [30]. Marattukalam et al. [56] found that the fraction of the crystalline phases for the SLMed bulk Zr_59.3_Cu_28.8_Al_10.4_Nb_1.5_ amorphous alloy increased with increasing laser powder. Lindwall et al. [57] found that the crystalline volume fraction in the HAZ for 3D-printed bulk Zr_59.3_Cu_28.8_Al_10.4_Nb_1.5_ amorphous alloy composites increased with the total energy input.

As shown in Figure 10, the local Avrami exponent for the present amorphous alloy composites is between 2~4, indicating the diffusion-controlled nucleation/growth mechanism [58]. In light of diffusion-controlled growth theory [59], 1 ˂ n ˂ 1.5 indicates a growth of particles of appreciable initial volume; n = 1.5 means a growth of particles with a nucleation rate close to zero; 1.5 ˂ n ˂ 2.5 implies a growth of particles with a decreasing nucleation rate; n = 2.5 indicates a growth of particles with a constant nucleation rate; and n > 2.5 corresponds to the growth of particles with an increasing nucleation rate. As shown in Figure 10, the dependences of the n(α) on the α for the studied powder and 3D-printed bulk samples are similar to each other at studied heating rates. When 0.05 ˂ α ˂ 0.6, n(α) > 2.5, it indicates the growth of particles with an increasing nucleation rate. When 0.6 ˂ α ˂ 0.95 and 0 ˂ α ˂ 0.05, 1.5 ˂ n(α) ˂ 2.5, it implies a growth of particles with a decreasing nucleation rate. If 0 ≤ α ≤ 0.95 is considered, the local Avrami exponent n(α) at different heating rates and averaged n(α) are listed in Table 4. As seen, the n(α) and averaged n(α) are almost the same as each other. The averaged n(α) is 2.6 for the 3D bulk samples printed under 15 and 15.6 J/mm^3^, indicating the growth of particles with an increasing nucleation rate. The averaged n(α) is 2.5 for the other cases, which implies the growth of particles with a constant nucleation rate. Wu et al. [60] investigated the crystallization path and non-isothermal kinetics of the Zr_59.5_Cu_14.4_Ni_11.6_Al_9.7_Nb_4.8_ metallic glass and found that the Avrami exponent is 3.2 for the metastable icosahedral quasicrystalline, 3.9 for Ni-containing phases, and 2.9 for Cu-containing phases, respectively. This indicates that the metastable icosahedral quasicrystalline and Ni-containing phases are interface-controlled growth, while the Cu-containing phases are diffusion-controlled growth. Ouyang et al. [20] found that the n values of Zr_55_Cu_30_Ni_5_Al_10_ and Zr_60.14_Cu_22.31_Fe_4.85_Al_9.7_Ag_3_ are 2~3 at the early stage of isothermal crystallization, indicating that the crystallization in the early stage is primarily controlled by a three-dimensional diffusion-controlled crystal growth mechanism in absence of nucleation. The n values for both alloys increase from 3 to 6 in the later crystallization stage, meaning an increase in the nucleation rate.

## 5. Conclusions

A series of Zr-Cu-Al-Co bulk amorphous alloy composites were 3D-printed under different conditions. Their crystallization behaviors were investigated, and the results are summarized as follows.

There are two phases, i.e., Cu_10_Zr_7_ and CuZr_2_ phases for the gas-atomized powder and the 3D-printed bulk samples. Both volume fraction and size of Cu_10_Zr_7_ and CuZr_2_ phases are larger for the 3D-printed samples than for the gas-atomized powders. In addition, both volume fraction and size of Cu_10_Zr_7_ and CuZr_2_ phases increase with increasing energy density.

The dependence of volume fraction on energy density can be fitted as *y* = 6.983 + 0.451exp(*x*/8.01) for Cu_10_Zr_7_ and *y* = 0.44 + 0.979exp(*x*/10.57) for CuZr_2_, respectively. The growth rate of volume fraction is larger for CuZr_2_ than for Cu_10_Zr_7_. However, the dependence of size on energy density can be fitted as *y* = 5.187 + 2.117*x* for Cu_10_Zr_7_ and *y* = 4.515 + 1.347*x* for CuZr_2_, respectively. The growth rate of size is larger for Cu_10_Zr_7_ than for CuZr_2_.

The characteristic temperatures including *T*_g_, *T*_x_, and *T*_p_ are close to each other for the gas-atomized powder and the 3D-printed bulk samples at corresponding heating rates. The Δ*T*_x_ is larger for the gas-atomized powders than for the 3D-printed bulk samples. The Δ*H*_x_ decreases with increasing energy density. The dependence of Δ*H*_x_ on amorphous content can be fitted as *y* = 12.513 + 6.609 × 10^−12^exp(*x*/3.276).

The sequence of the activation energies is *E*_g_ > *E*_x_ > *E*_p_ for the gas-atomized powder, while is *E*_x_ > *E*_p_ > *E*_g_ for the 3D-printed bulk samples. Moreover, the *E*_g_ is larger for the gas-atomized powder than for the 3D-printed bulk samples. Both *E*_x_ and *E*_p_ are larger for the 3D-printed bulk samples than for the gas-atomized powder.

The *E*_α_ decreases with increasing α for the gas-atomized powder and the 3D-printed bulk samples. In addition, the *E*_α_ is larger for the 3D-printed bulk samples than for the gas-atomized powder at the corresponding α. The dependence of the n(α) on the α is similar with each for the studied powder and the 3D-printed bulk samples at studied heating rates. When 0.05 ˂ α ˂ 0.6, n(α) > 2.5, it indicates the growth of particles with an increasing nucleation rate. When 0.6 ˂ α ˂ 0.95 and 0 ˂ α ˂ 0.05, 1.5 ˂ n(α) ˂ 2.5, it implies a growth of particles with decreasing nucleation rate.

## Figures and Tables

**Table 1:** Detailed SLM parameters.

Laser Power (W)	Scanning Velocity (mm/s)	Layer Thickness (mm)	Hatch Spacing (mm)	Energy Density (J/mm^3^)
75	2000	0.08	0.03	15.6
75	1500	0.08	0.03	20.8
90	2500	0.1	0.03	12
90	2000	0.1	0.03	15
90	1500	0.1	0.03	20

**Table 2:** Fraction and size for Cu_10_Zr_7_ and CuZr_2_ phases.

Energy Density (J/mm^3^)	0	12	15	15.6	20	20.8
Cu_10_Zr_7_ (Vol.%)	7.5	8.7	9.8	10.6	12.3	13.1
CuZr_2_ (Vol.%)	1.5	3.0	4.5	5.3	6.6	7.6
Cu_10_Zr_7_ (nm)	4.4	32.0	35.7	40.5	46.7	48.4
CuZr_2_ (nm)	5.2	17.5	25.4	27.4	31.4	32.5

**Table 3:** Crystallization enthalpy Δ*H*_x_, undercooled liquid region Δ*T*_x_, activation energies for glass transition *E*_g_, onset crystallization *E*_x_, and crystallization peak *E*_p_, respectively.

Power Density (J/mm^3^)	0	12	15	15.6	20	20.8
Δ*H*_x_ (J/g)	20.2	15.6	14.5	13.2	13.0	12.6
Δ*T*_x_ (K)	45.8	35.3	35.4	36.5	34.8	38.4
*E*_g_ (kJ/mol)	523.6	374.8	368.1	356.1	370.5	365.4
*E*_x_ (kJ/mol)	355.5	602.3	482.0	384.7	506.8	482.9
*E*_p_ (kJ/mol)	306.9	564.0	446.3	367.2	475.2	446.3

**Table 4:** Local Avrami exponent n(α) at different heating rates and averaged n(α) for 0 ≤ α ≤ 0.95.

Power Density (J/mm^3^)	0	12	15	15.6	20	20.8
10 K/min	2.5	2.4	2.7	2.7	2.5	2.6
20 K/min	2.5	2.5	2.7	2.6	2.5	2.5
30 K/min	2.5	2.5	2.6	2.5	2.4	2.5
40 K/min	2.4	2.5	2.5	2.4	2.5	2.5
Everaged n(α)	2.5	2.5	2.6	2.6	2.5	2.5

## Data Availability

The original contributions presented in this study are included in the article. Further inquiries can be directed to the corresponding authors.

## References

[1] Prabhu Y., Vincent S., Bhatt J. (2020). Thermodynamic modelling to optimize glass forming composition in multicomponent Zr-Cu-Co-Al system. Mater. Today Proc..

[2] Han K.M., Jiang H., Wang Y.M., Qiang J.B., Yu C.Y. (2021). Antimicrobial Zr-based bulk metallic glasses for surgical devices ap-plications. J. Non-Cryst. Solids.

[3] Wada T., Zhang T., Inoue A. (2003). Formation and high mechanical strength of bulk glassy alloys in Zr-Al-Co-Cu System. Mater. Trans..

[4] Zhang T., Inoue A. (2004). Formation, thermal and mechanical properties of bulk glassy alloys in Zr–Al–Co and Zr–Al–Co–Cu systems. Mater. Sci. Eng. A.

[5] Wang Z., Ketov S.V., Sun B.A., Chen C.L., Churyumov A.Y., Louzguine-Luzgin D.V. (2016). Eutectic crystallization during fracture of Zr–Cu–Co–Al metallic glass. Mater. Sci. Eng. A.

[6] Han K., Qiang J., Wang Y., Häussler P. (2017). Zr-Al-Co-Cu bulk metallic glasses for biomedical devices applications. J. Alloy. Compd..

[7] Kuo C., Huang J., Li J., Jang J., Lin C., Nieh T. (2013). Effects of B2 precipitate size on transformation-induced plasticity of Cu–Zr–Al glassy alloys. J. Alloy. Compd..

[8] Wu Y., Xiao Y., Chen G., Liu C.T., Lu Z. (2010). Bulk metallic glass composites with transformation-mediated work-hardening and ductility. Adv. Mater..

[9] Yang C., Zhang C., Xing W., Liu L. (2018). 3D printing of Zr-based bulk metallic glasses with complex geometries and enhanced catalytic properties. Intermetallics.

[10] Wu M.-W., Ni K., Lei Y., Xiong X.-X., Chuang Y.-T., Lin Q.-E., Wang P., Ramasamy P., Eckert J. (2023). Mechanical behavior of CuZrAl metallic glass scaffolds fabricated by selective laser melting. Mater. Lett..

[11] Yang C., Ouyang D., Zhang L., Zhang Y., Tong X., Ke H., Chan K., Wang W. (2024). The enhancement of damage tolerance of 3D-printed high strength architected metallic glasses by unit cell shape design. Addit. Manuf..

[12] Aliyu A.A.A., Panwisawas C., Shinjo J., Puncreobutr C., Reed R.C., Poungsiri K., Lohwongwatana B. (2023). Laser-based additive manufacturing of bulk metallic glasses: Recent advances and future perspectives for biomedical applications. J. Mater. Res. Technol..

[13] Wu W., Li X., Liu Q., Fuh J.Y.H., Zheng A., Zhou Y., Ren L., Li G. (2022). Additive manufacturing of bulk metallic glass: Principles, materials and prospects. Mater. Today Adv..

[14] Luo H., Fan A., Liao W., Du Y. (2024). Effect of laser power on the structure and wear performance of laser additively manufactured Cu_45_Zr_45_Al_6_Ti_4_ metallic glass coating. Surf. Coat. Technol..

[15] Liu H., Jiang Y., Yang D., Jiang Q., Yang W. (2023). Pores and cracks in the metallic glasses prepared by laser powder bed fusion. J. Mater. Res. Technol..

[16] Pauzon C., Daudin R., Robaut F., Berthomé G., Blandin J.-J. (2023). Laser powder bed fusion spatters of Zr-Cu-Al-Nb metallic glass. J. Alloy. Compd..

[17] Rodríguez-Sánchez M., Sadanand S., Ghavimi A., Busch R., Tiberto P., Ferrara E., Barrera G., Thorsson L., Wachter H., Gallino I. (2024). Relating laser powder bed fusion process parameters to (micro)structure and to soft magnetic behaviour in a Fe-based bulk metallic glass. Materialia.

[18] Li B., Yakubov V., Nomoto K., Ringer S.P., Gludovatz B., Li X., Kruzic J.J. (2024). Superior mechanical properties of a Zr-based bulk metallic glass via laser powder bed fusion process control. Acta Mater..

[19] Frey M., Wegner J., Barreto E.S., Ruschel L., Neuber N., Adam B., Riegler S.S., Jiang H.-R., Witt G., Ellendt N. (2023). Laser powder bed fusion of Cu-Ti-Zr-Ni bulk metallic glasses in the Vit_101_ alloy system. Addit. Manuf..

[20] Ouyang D., Zhang P., Zhang C., Liu L. (2021). Understanding of crystallization behaviors in laser 3D printing of bulk metallic glasses. Appl. Mater. Today.

[21] Pacheco V., Karlsson D., Marattukalam J.J., Stolpe M., Hjörvarsson B., Jansson U., Sahlberg M. (2020). Thermal stability and crystallization of a Zr-based metallic glass produced by suction casting and selective laser melting. J. Alloy. Compd..

[22] Ouyang D., Li N., Liu L. (2018). Structural heterogeneity in 3D printed Zr-based bulk metallic glass by selective laser melting. J. Alloy. Compd..

[23] Zhang P., Zhang C., Pan J., Ouyang D., Liu L. (2023). Toughening additive manufactured Zr-based bulk metallic glass composites by martensite phase transformation. J. Mater. Sci. Technol..

[24] Gao X., Liu Z., Li J., Liu E., Yue C., Zhao K., Yang G. (2020). Selective laser melting of CuZr-based metallic glass composites. Mater. Lett..

[25] Kozachkov H., Kolodziejska J., Johnson W.L., Hofmann D.C. (2013). Effect of cooling rate on the volume fraction of B2 phases in a CuZrAlCo metallic glass matrix composite. Intermetallics.

[26] Cai A., Zhou G., Li P., Ding D., An Q., Li Y., Yang Q., Mao H. (2023). Mechanical, wetting and corrosion properties of a Zr-based amorphous alloy composite consolidated by spark plasma sintering. J. Non-Cryst. Solids.

[27] Han X., Kaban I., Orava J., Cheng Q., Sun Y.H., Soldatov I., Zimmermann M.V., Song K., Nielsch K. (2022). Phase-formation maps of CuZrAlCo metallic glass explored by in situ ultrafast techniques. Acta Mater..

[28] Lan S., Wu Z., Wei X., Zhou J., Lu Z., Neuefeind J., Wang X.-L. (2018). Structure origin of a transition of classic-to-avalanche nucleation in Zr-Cu-Al bulk metallic glasses. Acta Mater..

[29] Wang C., Schmelzer J.W., Zhang L., Zhang L., Wang L., Schick C., Gao Y., Zhao B. (2024). Effect of cooling rate on the crystallization behaviors of Mg_65_Zn_30_Ca_5_ metallic glass composites. Intermetallics.

[30] Yang Z., Markl M., Körner C. (2024). Comprehensive numerical investigation of laser powder bed fusion process conditions for bulk metallic glasses. Addit. Manuf..

[31] Sohrabi N., Ivas T., Jhabvala J., Schawe J.E., Löffler J.F., Ghasemi-Tabasi H., Logé R.E. (2024). Quantitative prediction of crystallization in laser powder bed fusion of a Zr-based bulk metallic glass with high oxygen content. Mater. Des..

[32] Kissinger H.E. (1956). Variation of peak temperature with heating rate in differential thermal analysis. J. Res. Natl. Bur. Stand..

[33] Bing L., Li Y.H., Yang K., Li J.S., Fan X.H. (2016). Effect of yttrium addition on the non-isothermal crystallization kinetics and fragility of Cu-Zr-Al bulk metallic glass. Thermochim. Acta.

[34] Zhu M., Li J., Yao L., Jian Z., Chang F., Yang G. (2013). Non-isothermal crystallization kinetics and fragility of (Cu_46_Zr_47_Al_7_)97Ti_3_ bulk metallic glass investigated by differential scanning calorimetry. Thermochim. Acta.

[35] Cai A., Li P., Ding D., An Q., Zhou G., Yang Q., Lin Y., Mao H. (2022). Crystallization behavior of a series of Zr-based metallic glasses. Thermochim. Acta.

[36] An Q., Zhou G., Cai A., Li P., Ding D., Zhou G., Yang Q., Mao H. (2022). Effect of Ti and Al ratio on glass forming ability and crystallization behavior of Zr-Cu-Al-Ti alloy powders. Thermochim. Acta.

[37] Cai A., Li P., Ding D., An Q., Zhou G., Li Y., Mao H. (2023). Preparation and crystallization behavior of Cu-Zr-Ti amorphous composite powders. J. Non-Cryst. Solids.

[38] Cai A., Li P., Ding D., An Q., Zhou G., Yang Q., Lin Y., Mao H. (2022). Crystallization kinetics of Cu_50_Zr_40_Ti_10_ amorphous powder. Thermochim. Acta.

[39] Lu W., Yan B., Huang W.-H. (2005). Complex primary crystallization kinetics of amorphous Finemet alloy. J. Non-Cryst. Solids.

[40] Cai A., Zhou G., Ding D., Wu H., An Q., Zhou G., Yang Q., Li P. (2022). Effect of Ti addition on crystallization behavior of a Zr-based bulk metallic glass. Thermochim. Acta.

[41] Yang Z., Al-Mukadam R., Stolpe M., Markl M., Deubener J., Körner C. (2021). Isothermal crystallization kinetics of an industrial-grade Zr-based bulk metallic glass. J. Non-Cryst. Solids.

[42] Cui J., Li J., Wang J., Kou H., Qiao J., Gravier S., Blandin J. (2014). Crystallization kinetics of Cu_38_Zr_46_Ag_8_Al_8_ bulk metallic glass in different heating conditions. J. Non-Cryst. Solids.

[43] Gao Q., Jian Z.Y., Xu J.F., Zhu M., Chang F.G., Han A.M. (2016). Crystallization kinetics of the Cu_50_Zr_50_ metallic glass under iso-thermal conditions. J. Solid State Chem..

[44] Qiao J., Pelletier J. (2012). Isochronal and isothermal crystallization in Zr_55_Cu_30_Ni_5_ Al_10_ bulk metallic glass. Trans. Nonferrous Met. Soc. China.

[45] Sohrabi S., Gholamipour R. (2021). Effect of Nb minor addition on the crystallization kinetics of Zr-Cu-Al-Ni metallic glass. J. Non-Cryst. Solids.

[46] Pauly S., Das J., Mattern N., Kim D.H., Eckert J. (2009). Phase formation and thermal stability in Cu–Zr–Ti(Al) metallic glasses. Intermetallics.

[47] Qiao J., Pelletier J. (2011). Crystallization kinetics in Cu_46_Zr_45_Al_7_Y_2_ bulk metallic glass by differential scanning calorimetry (DSC). J. Non-Cryst. Solids.

[48] Blazquez J., Conde C., Conde A. (2005). Non-isothermal approach to isokinetic crystallization processes: Application to the nanocrystallization of HITPERM alloys. Acta Mater..

[49] Sun N., Liu X., Lu K. (1996). An explanation to the anomalous avrami exponent. Scr. Mater..

[50] Arroyave A., Eagar T.W., Kaufman L. (2003). Thermodynamic assessment of the Cu-Ti-Zr system. J. Alloys Compd..

[51] Sohrabi N., Schawe J.E.K., Jhabvala J., Löffler J.F., Logé R.E. (2021). Critical crystallization properties of an industrial-grade Zr-based metallic glass used in additive manufacturing. Scr. Mater..

[52] Kim J., Lee D., Shin S., Lee C. (2006). Phase evolution in Cu_54_Ni_6_Zr_22_Ti_18_ bulk metallic glass Nd: YAG laser weld. Mater. Sci. Eng. A.

[53] Sun H., Flores K. (2008). Laser deposition of a Cu-based metallic glass powder on a Zr-based glass substrate. J. Mater. Res..

[54] Ouyang D., Xing W., Li N., Li Y., Liu L. (2018). Structural evolutions in 3D-printed Fe-based metallic glass fabricated by selective laser melting. Addit. Manuf..

[55] Lindwall J., Lundbäck A., Marattukalam J.J., Ericsson A. (2022). Virtual Development of Process Parameters for Bulk Metallic Glass Formation in Laser-Based Powder Bed Fusion. Materials.

[56] Marattukalam J.J., Pacheco V., Karlsson D., Riekehr L., Lindwall J., Forsberg F., Jansson U., Sahlberg M., Hjörvarsson B. (2020). Development of process parameters for selective laser melting of a Zr-based bulk metallic glass. Addit. Manuf..

[57] Lindwall J., Ericsson A., Marattukalam J.J., Hassila C.-J., Karlsson D., Sahlberg M., Fisk M., Lundbäck A. (2022). Simulation of phase evolution in a Zr-based glass forming alloy during multiple laser remelting. J. Mater. Res. Technol..

[58] Christian J.W. (1965). The Theory of Transformation in Metals and Alloys.

[59] Henderson D.W. (1979). Experimental analysis of non-isothermal transformations involving nucleation and growth. J. Therm. Anal. Calorim..

[60] Wu Y., Li B., Zhu Y., Yuan X., Yan T., Zhang H., Fu H., Zhang H., Zhang L. (2024). Crystallization path and non-isothermal kinetics of the Zr_59.5_Cu_14.4_Ni_11.6_Al_9.7_Nb_4.8_ metallic glass under different heating rates. Scr. Mater..

